# Where America Leads Great Britain

**Published:** 1917-04-28

**Authors:** 


### WHERE AMERICA LEADS GREAT BRITAIN.

It is a remarkable fact, but it is true, that in Great Britain medical service and hospital treatment, for four decades at least, have been so ill organised and unmethodical, that they exclude the British middle classes, and it would be difficult to find any country where all classes except the very rich and the relatively poor have been left without adequate facilities for the treatment of medical and surgical ailments to the extent they have been in the Motherland. In America, when a member of the family, or the breadwinner, meets with an accident or is stricken by disease, he has his choice of several excellent systems of pay hospital accommodation where he can procure hospital care, the best medical or surgical treatment, including necessary operations, and everything which his case may need to expedite recovery, for an inclusive fee well within his means. Further, paying wards and accommodation for paying patients are so organised as to place facilities within reach of the patients of all classes to obtain the best medical treatment when their cases require the aid of a specialist, or whatever the nature of their ailment may be.

We made it our business last autumn to inspect the quarters and accommodation placed at the disposal of middle-class patients willing to pay, on the American system, inclusive fees for the accommodation and treatment they were in need of. Everywhere we found evidence of a well-developed system whereby economy and efficiency could be secured, the confidence of the patient maintained, and his rapid recovery expedited by every means experience, science, and technical and diagnostic skill could combine to secure. Mothers, in suitable cases, could accompany their children, and stay with them in the pay department during their residence and treatment there. Patients with special diseases could go to a sanatorium or home hospital which confined its work entirely to such cases. Nothing that could add to the patient's comfort, or increase the confidence of himself and his friends in the management of the pay department or institution was omitted. The equipment, the nursing, the diets and service were all of the very best. Further, it was demonstrated by actual figures that the pay system, conducted on the American plan, like that which has prevailed for thirty years at the mother institution of pay hospitals in the United Kingdom, Fitzroy House, London, under efficient management, yields the best return to everybody concerned—*i.e.*, to the patient, the practitioner, and the managers of the pay hospital or paying wards, —whilst the patient's friends are well content to leave him in a pay institution which experience has led them to trust, where the smooth working of every detail consequently results.

